# To achieve 95-95-95 targets we must reach men and youth: High level of knowledge of HIV status, ART coverage, and viral suppression in the Botswana Combination Prevention Project through universal test and treat approach

**DOI:** 10.1371/journal.pone.0255227

**Published:** 2021-08-10

**Authors:** Refeletswe Lebelonyane, Pamela Bachanas, Lisa Block, Faith Ussery, Mary Grace Alwano, Tafireyi Marukutira, Shenaaz El Halabi, Michelle Roland, William Abrams, Gene Ussery, James A. Miller, Shahin Lockman, Tendani Gaolathe, Molly Pretorius Holme, Shannon Hader, Lisa A. Mills, Kathleen Wirth, Naomi Bock, Janet Moore

**Affiliations:** 1 Ministry of Health and Wellness, Gaborone, Botswana; 2 U.S. Centers for Disease Control and Prevention, Atlanta, GA, United States of America; 3 Northrop Grumman, Atlanta, GA, United States of America; 4 U.S. Centers for Disease Control and Prevention, Gaborone, Botswana; 5 Harvard T.H. Chan School of Public Health, Boston, MA, United States of America; 6 Botswana Harvard AIDS Institute Partnership, Gaborone, Botswana; 7 UNAIDS, Geneva, Switzerland; International AIDS Vaccine Initiative, UNITED STATES

## Abstract

**Background:**

Increasing HIV treatment coverage is crucial to reducing population-level HIV incidence.

**Methods:**

The Botswana Combination Prevention Project (BCPP) was a community randomized trial examining the impact of multiple prevention interventions on population-level HIV incidence and was conducted from October 2013 through June 2017. Home and mobile campaigns offered HIV testing to all individuals ≥ age 16. All identified HIV-positive persons who were not on antiretroviral therapy (ART) were referred to treatment and tracked to determine linkage to care, ART status, retention in treatment, and viral suppression

**Results:**

Of an estimated total of 14,270 people living with HIV (PLHIV) residing in the 15 intervention communities, BCPP identified 13,328 HIV-positive persons (93%). At study start, 10,703 (80%) of estimated PLHIV knew their status; 2,625 (20%) learned their status during BCPP, a 25% increase with the greatest increases occurring among men (37%) and youth (77%). At study start, 9,258 (65%) of estimated PLHIV were on ART. An additional 3,001 persons started ART through the study. By study end, 12,259 had initiated and were retained on ART, increasing coverage to 93%. A greater increase in ART coverage was achieved among men (40%) compared to women (29%). Of the 11,954 persons who had viral load (VL) test results, 11,687 (98%) were virally suppressed (HIV-1 RNA ≤400 copies/mL). Overall, 82% had documented VL suppression by study end.

**Conclusions:**

Knowledge of HIV-positive status and ART coverage increased towards 95–95 targets with universal testing, linkage interventions, and ART. The increases in HIV testing and ART use among men and youth were essential to reaching these targets.

**Clinical trial number:**

NCT01965470.

## Introduction

In 2014, the Joint United Nations Programme on HIV/AIDS (UNAIDS) set new HIV testing and treatment targets aimed at “reversing the AIDS epidemic” by 2020 and ending the AIDS epidemic by 2030 [1]. Shortly thereafter, the World Health Organisation (WHO) changed its guidelines to recommend universal treatment of all HIV-positive persons regardless of CD4 cell count or WHO stage of disease [2]. Several trials in southern and eastern African countries have evaluated the feasiblity of implementing universal HIV testing and treatment (UTT), achieving the UNAIDS 90-90-90 targets, and impacting HIV incidence at the population level [3].

In Botswana, where one of the trials was conducted, an estimated 22·8% of the adult population is living with HIV, the third highest HIV prevalence globally [4]. By 2015, Botswana had almost achieved the UNAIDS “90-90-90” targets for HIV diagnoses, treatment, and viral suppression [5, 6]. Even so, annual HIV incidence among adults remained high at 1·3% [4, 6].

The primary aim of the Botswana Combination Prevention Project (BCPP) was to determine if implementing highly-targeted interventions to reach persons missed by the robust national HIV testing programs and treatment programs could significantly decrease HIV incidence to a level below that already achieved in the country. Final results from BCPP showed a 30% reduction in annualized incidence in intervention compared to control communities [7]. Equally important aims of this study were to 1) determine the feasibility of exceeding 90-90-90 targets in a country with mature HIV testing and treatment programs, 2) determine if HIV-positive persons can be retained in the treatment cascade, and achieve viral load suppression (VLS), and 3) identify sub-populations who are not reaching 90-90-90 targets and determine if gaps can be closed. As more countries are approaching achievement of the 90-90-90 targets and setting new targets at 95-95-95 levels [8], it is important to document the feasibility of reaching these targets.

## Methods

### Study design

BCPP was a cluster-randomized HIV prevention trial to evaluate the impact of UTT on population-level HIV incidence [3, 6, 7]. The trial was conducted from October 2013 to June 2017 (with one gap of 6 months to refine data collection procedures) in 30 rural and peri-urban villages in eastern and northern Botswana. Each community had an average population size of approximately 6,000. Fifteen communities received the interventions and 15 matched control communities received standard of care. All HIV-positive individuals identified by BCPP in the intervention communities were followed over the course of the study to document their retention on treatment and achievement of VLS. Results presented in this paper are from sub-analyses of extensive programmatic data collected from participants in the 15 intervention communities only. Similar data were not collected in the control communities due to concern that data collection through follow-up of participants would be an intervention in itself and due to cost and lack of staffing.

### Study participants

Participants were 16 to 64 years old, community residents (defined as having spent at least 3 nights/month in the community), and Botswana citizens or spouses of citizens. Participants were identified through community case finding or HIV testing programs.

### Study interventions and data collection

#### Enumeration of households

Enumeration of households was used to derive an estimate of the number of HIV-positive persons residing in each community. Enumeration also allowed for interaction with household members about the study and helped to mobilize the community to participate in intervention activities. Google maps were used to identify plots in the study communities with residential and habitable structures. GPS coordinates were used to locate households. HIV counselors enumerated members for each household with an eligible informant present. Our goal was to enumerate 100% of households but we were only able to enumerate 75% of the households. We then extrapolated from the number of members found in enumerated households by age and sex to 25% of households that were not enumerated to derive an estimate of the number of residents in the 15 communities. Using the HIV prevalence detected in an earlier 20% baseline household survey sample adjusted for age and sex, we estimated the number of HIV-positive persons residing in the 15 communities [6].

#### Case finding, HIV testing, and referral to care

All HIV-positive participants in the 15 intervention communities were identified through assessment of their HIV status either in the home or in mobile venues. If persons determined to reside in households through enumeration were not at home at first contact, additional home visits were made for up to three visits. In communities where we were unable to identify the number of HIV-positive persons that our estimates suggested lived in the communities, we worked with community leaders to help identify where certain groups could be accessed. For example, our data suggested that men and youth were underrepresented in our HIV testing activities. By consulting with community leaders, we were able to determine venues where we could these find these groups and approach them about HIV testing. Rather than focusing on home-based testing, HIV testing counselors accessed places in the communities such as workplaces, markets, shabeens, farms, etc. where large groups of men and youth congregated.

All persons agreeing to participate completed an intake assessment with questions on demographics, prior HIV testing history and status, and treatment history if HIV-positive. Those reporting HIV-positive status were asked for documentation of status and treatment regimen if on ART. Persons who did not know their status or who did not have documentation of positive HIV status or of a negative HIV test within the preceding three months were offered HIV testing. Verbal consent for completing the intake assessment and HIV testing is the standard in Botswana, and the age of consent for testing is 16. Therefore, written consent was not required and verbal consent was documented by the interviewer/counselor on the electronic version of the form. Counselors followed the national testing algorithm of parallel HIV rapid tests (KHB, Shanghai Kehua Bio-Engineering Co Ltd, Shanghai, China and Unigold, Trinity Biotech Plc, Bray, Ireland). Persons identified through BCPP testing interventions or who already knew their HIV-positive status but were not on ART were provided with linkage to care interventions. Participants were given appointments to their local community clinics and sent a short message service appointment reminder. Participants were tracked through the clinic electronic medical records (EMR) and clinic registers. Those who did not keep their appointments were traced through home visits and phone calls.

Collection of intake information, provision of HIV testing, and tracking of participants were performed by trained lay counselors. All counselors were certified to conduct HIV testing through the Botswana Ministry of Health national training program and received extensive training in interviewing techniques and study procedures from BCPP. They also were provided with written scripts to help them explain study rationale, methods, and procedures to participants.

#### ART and retention in care

A key component of the BCPP intervention package was “early” ART for HIV-positive individuals. Standard of care was ART initiation at CD4 ≤ 350 or WHO stage III or IV from 2013 through 2016, while criteria for “early” ART in BCPP also included those with CD4 cell counts ≥ 350-<500cells/μL or CD4 ≥ 500 cells/μL and VL > 10,000 copies/μL. The use of high viral load (VL) as an additional eligibility criterion was based on the known association between VL and HIV transmission [9–11]. In mid-2016, the government of Botswana adopted UTT and all HIV-positive persons in both study arms were referred for ART, regardless of CD4 cell count or disease stage [12].

An additional treatment component implemented in intervention communities was rapid ART initiation [12]. ART was offered on the first clinic visit to patients assessed as medically stable. If abnormal laboratory test results were later returned, patients were recalled to the clinic and adjustments in medication were made if indicated.

All HIV-positive persons identified in BCPP whether newly or previously diagnosed were tracked through their electronic medical records (EMR) to determine initiation of ART and retention in care. Individuals who failed to keep clinic appointments were traced through phone calls and home visits by the clinic nurse or community counselor.

#### Viral load testing

VL testing was conducted at three months post-initiation of ART and every six months thereafter. VLS was defined as ≤400 copies/ml. Persons who missed VL appointments or were not tested by the clinics at the appropriate times were traced by phone calls by the clinic nurse and asked to return to the clinic for VL testing. Attempts to contact patients were limited to three phone calls.

#### Data management

Multiple data sources for BCPP interventions were combined into a single research database designed in Postgre Structured Query Language. All intake questionnaires and HIV test results were collected on encrypted handheld tablets by trained local lay counselors. Clinical information related to HIV treatment and laboratory results was obtained from the existing Ministry of Health and Welfare electronic medical records. Data from all sources were merged into a research database using Botswana’s individual unique national identifier (Omang) issued by the government to citizens at age 16 years. The Omang number allowed for tracking across time of all HIV-positive persons identified in BCPP from intake through the treatment cascade and prevented double counting of persons tested through different venues and persons getting care at multiple health facilities.

#### Analytic approach

Variables for analysis were defined as follows:

- The number of HIV-positive persons who knew their status prior to the start of BCPP was the sum of persons interviewed by BCPP in home or mobile venues who had documentation of HIV-positive status (either with a prior test result, health card, or ART prescription) or had an HIV-positive test date in the EMR.
- The total number of HIV-positive persons who knew their HIV status at study end included all persons who were assessed by BCPP who had documentation of an HIV-positive status or tested HIV-positive during the study period.
- Coverage of knowledge of HIV-positive status (1^st^ 90) was calculated by dividing the total number of HIV-positive persons identified by the estimated number of HIV-positive adults living in the 15 communities.
- The number of people on ART prior to the start of BCPP included those with documentation of being on ART (health cards, prescriptions, pill bottles) and/or via clinic EMR.
- The number of people on ART at study end was the sum of those who were alive and retained on ART. Retention was indicated by EMR documentation of an ART refill within the previous 4 months or a clinic appointment within the previous 6 months after ART initiation. Also, as VL testing is regularly conducted on all persons on ART, a VL result in the previous 18 months after initiation was also evidence of retention on ART.
- Coverage (2^nd^ 90) was derived by dividing the total number of HIV-positive individuals on ART at study end by the total number of HIV-positive persons identified by BCPP who were alive and knew their status.
- Viral suppression coverage (3^rd^ 90) was based on the last VL test result in the EMR recorded between January 2017 and June 2018. VL test results ≤400 copies/ml were classified as suppressed. Coverage was calculated using two different denominators: 1) number of HIV-positive persons identified through BCPP on ART, who were alive at study end, and had an available VL result; and 2) total number of HIV-positive persons identified whether on ART or not who were alive at study end.
- Population level viral suppression was computed by multiplying 1) the percentage of the estimated number of all HIV-positive persons who knew their HIV status by 2) the percentage of persons who knew their HIV status who were on ART, by 3) the percentage of persons on ART who were virally suppressed.

#### Analyses

SAS version 9.4 (SAS Institute, Cary, NC) was used for all statistical analyses. Descriptive frequencies were produced. The change in knowledge of HIV-positive status from the beginning of the study to the end of the study was measured by calculating the absolute difference overall and stratified by sex and age. This difference in knowledge at study start and study end was tested using the Rao-Scott Chi-square test, accounting for community level clustering. A logistic regression model was used to regress sex and age as an interaction term on knowledge of HIV-positive status to assess if there was an interaction effect present (F-value reported). The change in ART coverage from the beginning of the study to the end of the study was measured by calculating the absolute difference overall and stratified by sex and age. The Rao-Scott Chi-square test was used to test for a difference in the overall proportion that started ART by gender and by age. A logistic regression model was used to regress sex and age as an interaction term on starting ART by study end to assess if there was an interaction effect present (F-value reported). Unadjusted and adjusted prevalence ratio estimates describe the association between not being virally suppressed and sociodemographic factors using a log-binomial regression model, accounting for clustering of observations by community. All significant sociodemographic factors from the univariate analysis were included in the adjusted model. A type II error rate of α = 0.05 was used to determine statistical significance.

#### Ethics

The study was approved by the United States Centers for Disease Control and Prevention Institutional Review Board (Protocol #6475) and the Botswana Health Research and Development Committee (Institutional Review Board of the Botswana MoHW HPDME 13/18/1).

## Results

### Household enumeration and HIV-positive population estimate

A total of 27,351 plots were identified in the 15 intervention communities with 28,383 households that were residential and habitable (Google maps 2013 and census data 2011). Of the identified households, 21,152 (75%) were enumerated; and 45,636 study eligible household members were enumerated. For the 7,231 households that were not enumerated, we extrapolated from the number of members in the visited households to estimate the number of persons missed. We determined that 15,908 household members were missed and that approximately 61,544 study eligible persons resided in the 15 intervention communities. Using the HIV prevalence from an earlier BCPP baseline survey of 20% of randomly sampled households in the 15 communities and adjusted for age and sex [6] we estimated 14,270 (95% CI: 13,497, 15041) persons with HIV resided in the fifteen intervention communities.

### HIV assessment and testing in BCPP

Through home-based and mobile testing programs, BCPP interviewed 64,086 unique residents which exceeded the estimated number of residents in the intervention communities by 2,542, likely due to population growth and persons aging into the age range of participants over the course of the study. Of the residents interviewed, we assessed the HIV status of 61,655 unique residents; 2,431 (4%) of the individuals interviewed refused to report or provide documentation of their HIV status and/or refused HIV testing. Of the individuals assessed, 33,763 were found through the BCPP case finding/ home-based testing and 27,892 residents who had not been assessed in the home were found through mobile testing programs.

### Knowledge of positive status

**Fig 1:**
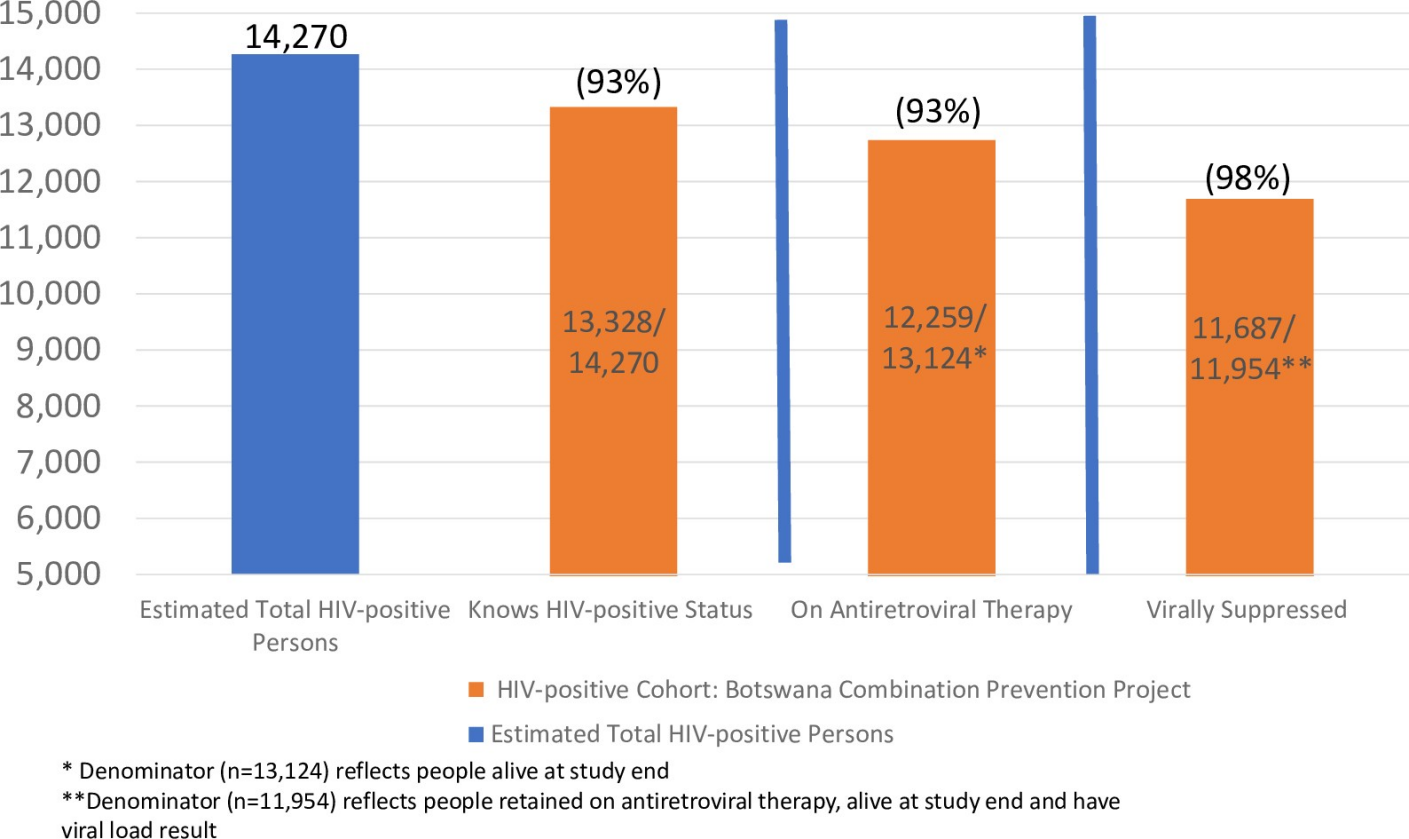
HIV care cascade among HIV-infected adults in 15 intervention communities.

**Fig 2:**
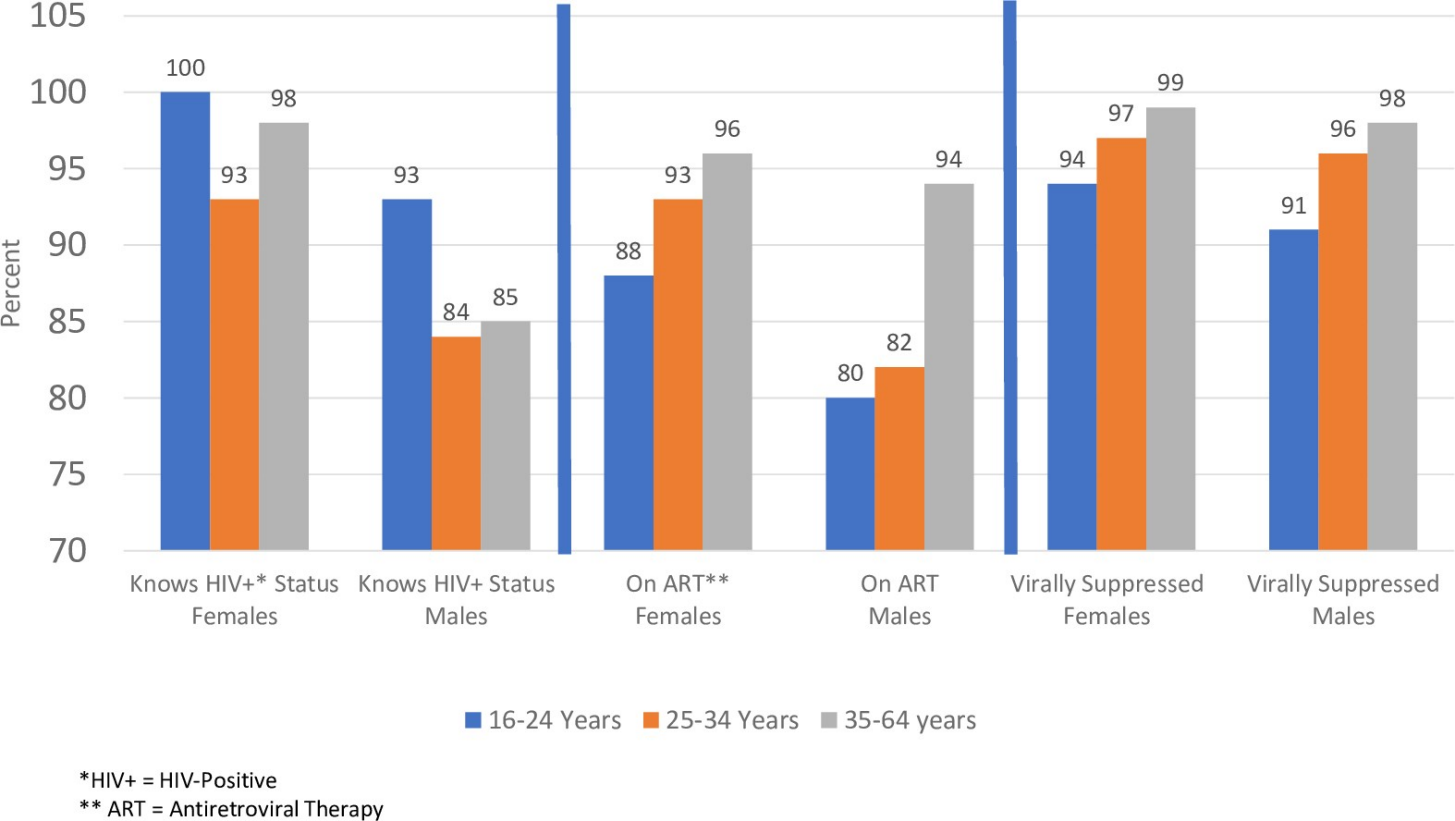
Knowledge of HIV-positive status, ART status, and viral suppression stratified by age and sex.

BCPP identified and enrolled 13,328 HIV-positive persons from study start to study end; 10,703 (80%) knew their HIV-positive status prior to enrollment in the study, 2,040 (15%) were diagnosed through BCPP testing interventions, and 585 (5%) persons learned their HIV-positive status during the study and were included in analyses but were not tested through the BCPP testing interventions. The 13,328 HIV-positive persons that BCPP identified was 93% (1^st^ UNAIDS 90) of the estimated 14,270 HIV-positive persons residing in the 15 intervention communities (Fig 1). More women than men knew their HIV-positive status across all age categories (Fig 2).

The percent change in knowledge of HIV-positive status from 10,703 persons at study start to 13,328 at study end was 25%, however, the increase varied significantly across age and sex (Table 1). A significantly greater percent change was observed among men (37%) than women (19%) (p <0.0001). A significantly greater percent change was found for younger persons; 77% for 16–24 years, 41% for 25–34, and 16% for 35–64. When we looked at the association between sex and age among those who learned their HIV-positive status, there was a significant interaction effect of sex and age (F-value = 15.11; p<0 .0004). Among women, the percent change in knowledge of status was greatest in those 16–24 years (73%) compared to those 25–34 years (27%) and 35 to 64 years (11%; p<0.0001). For men, however, the greatest percent change was among men 25–34 (114% increase) as compared to men 16–24 (89%) and men 35–64 (24%; p<0.0001).

**Table 1:** Increases in knowledge of HIV-positive status from study start to study end in the intervention communities by sex and age.

	Study Start	Study End	Absolute Difference	p-value*
	(n)	(n)		
**Women**	7,527	8,981	1,454	
16–24	493	854	361	
25–34	1,873	2,379	506	<0.0001
35–64	5,161	5,748	587	
**Men**	3,176	4,347	1,171	
16–24	131	248	117	
25–34	360	769	409	<0.0001
35–64	2,685	3,330	645	
**Total**	10,703	13,328	2,625	

n, number of participants

*Rao-Scott Chi-Square test for difference in knowledge at study start and study end; **Significant at α = .05

### ART coverage

Among the 13,328 HIV-positive persons identified through BCPP, 204 had died by study end. Of the 13,124 persons alive at study end, 12,259 or 93% (Fig 1) had documentation of being on ART, representing the 2^nd^ UNAIDS target. ART coverage at study end ranged from 92% in younger women (16–34) to 96% in women aged 35–64. Among men, coverage ranged from 81% in younger men (16–34) to 94% in older men (Fig 2).

At study start, 9,258 of identified HIV-positive persons were on ART. The number of persons on ART at study end had increased to 12,259, a 32% change (Table 2). A greater percent change in ART coverage was achieved among men (40%) compared to women (29%) (F-value = 61.89; p<0.0001). Also, a greater percent change in ART coverage was seen among younger than older people (F-value = 251.97, p<0.0001); 93% change among persons 16–24, 60% among persons 25–34, and 21% among persons 35–64. When we looked at the association between sex and age among those who started ART by study end, a significant interaction effect of sex and age was also observed; for women, a percent change from study start to study end of 99% was observed for those age 16–24, 46% for those 25–34, and 18% for women 35–64 (p<0.0001). A slightly different pattern was seen among men with a 73% percent change among men 16–24, an even greater percent change among men 25–34 (143%), and a lesser degree of change for men 35–64 (27%; p<0.0001).

**Table 2:** Increases in ART coverage from study start to study end in the intervention communities by sex and age.

	Study Start	Study End	Absolute Difference	p-value*
	(n)	(n)		
**Women**	6,488	8,384	1,896	
16–24	374	745	371	
25–34	1,494	2,182	688	<0.0001
35–64	4,620	5,457	837	
**Men**	2,770	3,875	1,105	
16–24	113	196	83	
25–34	257	624	367	<0.0001
35–64	2,400	3,055	655	
**Total**	9,258	12,259	3,001	

n, number of participants

*Rao-Scott Chi-Square test for difference in overall proportion; **Significant at α = .05

### Viral suppression

Of the 12,259 persons on ART at study end, 11,954 had current viral load results available in the EMR (within past 18 months) and of those 11,687 or 98% were virally suppressed (UNAIDS 3^rd^ 90; Fig 1). If the conservative assumption is made that persons who did not have a VL result were not currently virally suppressed (i.e., missing = failure), then the proportion of persons on ART who were virally suppressed drops to 95% (11,687/12,259). Viral suppression rates among persons on ART ranged from 94–99% across female age categories and 91–98% across male age categories (Fig 2).

For the HIV-positive persons identified by BCPP and alive at study end whether on ART or not (10,11), we found 1,473 who were not currently virally suppressed. We observed that 23.9% of those aged 16–24 were not virally suppressed and 15.4% of those aged 25–34 were not virally suppressed. The majority of people aged 35–49% were virally suppressed, with only 7.8% not virally suppressed. In an adjusted model, we examined characteristics of those not currently virally suppressed (Table 3). Controlling for age and all other factors, the proportion of men who were not virally suppressed was 1.49 times greater than women (95% CI: 1.36,1.62). Participants with primary or lower education were 0.71 times less likely to not be virally suppressed than those with secondary or higher education (95% CI: 0.63,0.80). Participants who were employed were 1.26 times more likely to not be virally suppressed than those unemployed (95% CI: 1.10,1.44). The proportion of participants who were not virally suppressed was 1.36 times greater among those who used alcohol compared to those who did not use alcohol (95% CI: 1.25, 1.47).

**Table 3:** Factors associated with no viral suppression at study end in the intervention communities, n = 13,124^1^.

Variable	Unsuppressed N (%)	Unadjusted PR	p-value	Adjusted PR^2^	p-value
		(95% CI)		(95% CI)	
**Sex**					
Women	842 (9.5)	ref	---	ref	---
Men	595 (14.0)	1.47 (1.36,1.59)	<0.001*	1.49 (1.36, 1.62)	<0.001*
**Marital status** ^**a**^					
Married	384 (10.1)	0.87 (0.74,1.02)	0.08	1.07 (0.93, 1.24)	0.38
Divorced/separated/widowed	37 (5.9)	0.51 (0.39,0.65)	<0.001*	0.92 (0.73, 1.16)	0.49
Single/never married	1,016 (11.7)	ref	---	ref	---
**Education** ^**b**^					
Primary/lower	383 (7.1)	0.52 (0.45, 0.61)	<0.001*	0.71 (0.63,0.80)	<0.001*
Secondary or higher	1,052 (13.6)	ref	---	ref	---
**Employment** ^**c**^					
Employed	682 (12.6)	1.30 (1.15,1.46)	0.001*	1.26 (1.10, 1.44)	<0.001*
Unemployed	747 (9.7)	ref	---	ref	---
**Alcohol use** ^**d**^					
Yes	446 (15.5)	1.61 (1.46,1.77)	<0.001*	1.36 (1.25, 1.47)	<0.0001*
No	979 (9.6)	ref	---	ref	---

^1^Sample includes only HIV-positive persons identified by BCPP and alive at study end, regardless of ART status.

^2^Adjusted prevalence ratio describes the association between not being viral suppressed and sociodemographic factors, after adjusting for age.

*Significant at α = .05

^**a**^missing n = 3

^**b**^missing n = 13

^**c**^missing n = 34

^**d**^missing n = 62

## Discussion

Despite relatively high rates of HIV testing and ART coverage at baseline in these 15 communities, significant increases were observed from study start to end for both proportion of people who knew their HIV-positive status (25%) and proportion of PLHIV on ART (32%). At study end, coverage of knowledge of status, on ART, and VLS were 93%, 93%, and 95% respectively. Population level viral suppression was 82%. Similar findings have been reported for two of the other large UTT trials focused on reaching 90-90-90 targets and reducing HIV incidence [13–15]. Together, these trials suggest that the new ambitious UNAIDS targets of 95-95-95 are feasible and that the interventions used in these trials may make important contributions to reductions in incidence.

Although overall increases in knowledge of HIV-positive status and number of PLHIV on ART were impressive, careful examination of program data revealed that the greatest gains were seen among individuals in the sex and age strata which had lower levels of coverage at study start, specifically women 16–24 years of age and men under 35 years of age. We speculate that our targeted efforts to reach men and youth in the community, whether at the workplace, shebeens, markets, cattle posts, or technical schools, had a substantial effect on the increases in knowledge of HIV status in these groups. This targeted outreach was possible in part because BCPP had population-based estimates of the number of HIV-positive persons who should have been identified based on age and sex [8]; thus, the gaps were apparent and could be addressed. Not every country will have local estimates of HIV incidence or prevalence. Nonetheless, the literature has been consistent in recognizing that men overall and young women are less likely to know their HIV-positive status and implementing strategic programs to overcome these gaps in generalized epidemics will be key to reaching the first 90 [16–19].

In addition to age and sex, we found additional sociodemographic characteristics associated with not being virally suppressed. In addition to being young and male, having more education, being employed, and using alcohol were associated with not being virally suppressed. During implementation of BCPP, we found that targeting testing to specific venues was helpful for finding men, youth, and employed persons for testing. It is important that we also determine the best methods for delivering ART and monitoring viral suppression among these groups. Employed persons are likely to need services that are near their work and available at times before or after work. Also given that men and youth are less frequent users of health facilities than women and older persons, other community-based venues for delivering ART and monitoring health outcomes may increase uptake of services. Citizen identification number along with the national HIV EMR enabled staff to follow HIV-positive individuals from community testing to health facility treatment and to avoid double counting of participants. WHO has begun to address the need to track patients beyond the testing program or ART initiating clinic with their recommendations for case-based surveillance [20]. These call for “the adoption or expansion of unique patient identifiers to link individual patient records within facilities, programs and between different health services” and ultimately for a national EMR system. BCPP clearly benefited from Botswana’s early adoption of these practices.

This study had several limitations. First, the analyses presented here are from the intervention arm only and we do not have comparable data from the control communities. The extensive programmatic data collected in the intervention arm was not possible in control communities because of costs and availability of staff. Equally important, however, implementing extensive data collection in control communities would have acted as an intervention in itself detracting from and blurring the line between intervention and control communities. Consequently, we were unable to attribute gains and outcomes in the intervention communities to our particular interventions. However, data from a sample of communities comparing ART coverage at study end in a sample of three intervention and three control communities showed significantly higher knowledge of HIV status and ART uptake in intervention communities [21]. Another limitation of the study was that the estimation of the number of HIV-positive persons living in these communities was derived by extrapolating to non-enumerated households and might have resulted in under or overestimation of coverage of knowledge of status. Given that ART use and viral suppression were directly measured rather than estimated, it is likely that they provide more stable, reliable measures of 95-95-95 attainment.

The success of BCPP in implementing UTT and achieving a high rate of VLS in a country with high HIV burden but also high achievement at baseline of UNAIDS targets demonstrates the potential for continued progress toward reversing the AIDS epidemic as countries get closer to their goals. The findings also highlight the feasibility of reaching men and youth and their contribution to achieving UNAIDS targets.

## Supporting information

S1 File (DOCX)
