# Supplementary material for: To achieve 95-95-95 targets we must reach men and youth: High level of knowledge of HIV status, ART coverage, and viral suppression in the Botswana Combination Prevention Project through universal test and treat approach
Source: PLoS One. 2021 Aug 10;16(8):e0255227. doi: 10.1371/journal.pone.0255227 (PMC8354449; doi:10.1371/journal.pone.0255227)
Supplement: S1 File — (DOCX) [file pone.0255227.s001.docx]

To whom it may concern:

The Botswana Combination Prevention Project (BCPP) has publicly released its dataset!

Links to BCPP data:

[Botswana Combination Prevention Project (BCPP) - Public Release Data | Data | Centers for Disease Control and Prevention (cdc.gov)](https://data.cdc.gov/Global-Health/Botswana-Combination-Prevention-Project-BCPP-Publi/qcw5-4m9q)

Direct weblink: <https://data.cdc.gov/Global-Health/Botswana-Combination-Prevention-Project-BCPP-Publi/qcw5-4m9q>

You will see that the data files are available as “BCPP Datasets.zip” under “Download this Resource”.

Under the section “About this Dataset”, you will see “Show More” 🡪 click on this and you will find the supporting attachments, such as the data dictionaries, data request form, etc.

If you need assistance or wish to request data directly, please contact:

Faith Ussery, MPH at [inh3@cdc.gov](mailto:inh3@cdc.gov)
